# Olfactory Memory Impairment Differs by Sex in a Rodent Model of Pediatric Radiotherapy

**DOI:** 10.3389/fnbeh.2018.00158

**Published:** 2018-08-02

**Authors:** Emma C. Perez, Shaefali P. Rodgers, Taeko Inoue, Steen E. Pedersen, J. Leigh Leasure, M. Waleed Gaber

**Affiliations:** ^1^Behavioral Neuroscience Lab, Department of Psychology, University of Houston, Houston, TX, United States; ^2^Texas Children’s Cancer Center, Department of Pediatrics, Dan L. Duncan Cancer Center, Baylor College of Medicine, Houston, TX, United States; ^3^Department of Molecular Physiology and Biophysics, Baylor College of Medicine, Houston, TX, United States; ^4^Department of Physiology and Biochemistry, Ross University School of Medicine, Roseau, Dominica; ^5^Department of Biology and Biochemistry, University of Houston, Houston, TX, United States

**Keywords:** social odor, olfactory recognition memory, radiotherapy, sex differences, cranial irradiation

## Abstract

Although an effective treatment for pediatric brain tumors, cranial radiation therapy (CRT) damages surrounding healthy tissue, thereby disrupting brain development. Animal models of pediatric CRT have primarily relied on visual tasks to assess cognitive impairment. Moreover, there has been a lack of sex comparisons as most research on the cognitive effects of pediatric CRT does not include females. Therefore, we utilized olfaction, an ethologically relevant sensory modality, to assess cognitive impairment in an animal model of CRT that included both male and female mice. Specifically, we used the novel odor recognition (NOdorR) task with social odors to test recognition memory, a cognitive parameter that has been associated with olfactory neurogenesis, a form of cellular plasticity damaged by CRT. In addition to odor recognition memory, olfactory ability or discrimination of non-social and social odors were assessed both acutely and 3 months after CRT. Magnetic resonance imaging (MRI) and histology were performed after behavioral testing to assess long-term damage by CRT. Long-term but not acute radiation-induced impairment in odor recognition memory was observed, consistent with delayed onset of cognitive impairment in human patients. Males showed greater exploration of social odors than females, but general exploration was not affected by irradiation. However, irradiated males had impaired odor recognition memory in adulthood, compared to non-irradiated males (or simply male controls). Female olfactory recognition memory, in contrast, was dependent on estrus stage. CRT damage was demonstrated by (1) histological evaluation of olfactory neurogenesis, which suggested a reduction in CRT versus control, and (2) imaging analyses which showed that the majority of brain regions were reduced in volume by CRT. Specifically, two regions involved in social odor processing (amygdala and piriform cortex) were damaged by cranial irradiation in males but not females, paralleling olfactory recognition findings.

## Introduction

Cranial radiation therapy is a successful treatment for cancer of the central nervous system, the second leading type of cancer in children (American Cancer Society, 2012). CRT can have detrimental effects on intellectual functioning, including memory, executive function, verbal fluency, visual-motor function, and IQ (Spiegler et al., 2004). Brain tumor survivors treated with CRT demonstrate more pronounced cognitive dysfunctions than those treated without CRT (Copeland et al., 1985; Ullrich and Embry, 2012). These “late effects” typically manifest after a few years post-CRT, and younger age at the time of radiation treatment is associated with greater cognitive impairment later in life (Ullrich and Embry, 2012). Moreover, there have been reports of greater detrimental effects in female pediatric survivors of radiotherapy, such as physical growth (Craig et al., 1999; Darzy and Shalet, 2006) and psychosocial outcomes (Hudson, 2003; Pui et al., 2003).

In the last two decades, pre-clinical or animal models of CRT have largely focused on measuring cognitive changes associated with impairment in hippocampal cell proliferation and neurogenesis (Raber et al., 2004; Rola et al., 2004; Wong-Goodrich et al., 2010; Roughton et al., 2012), a cellular impairment that persists after CRT (Rodgers et al., 2016). The majority of behavioral CRT research employs standard tasks, such as spatial learning in the Morris water maze and novel object recognition, robust tests of learning and memory. Using a pediatric model of CRT, we have recently demonstrated executive function impairment observable at 3 and 12 months post-CRT using the five-choice serial reaction time task (Sahnoune et al., 2018). Numerous other studies on the long-term effects of CRT demonstrate subtle cognitive impairments, such as spatial memory and/or object recognition memory impairments (Rola et al., 2004; Wong-Goodrich et al., 2010; Rao et al., 2011; Warrington et al., 2012) which have been correlated with impaired hippocampal cell proliferation and neurogenesis (Raber et al., 2004; Rola et al., 2004; Wong-Goodrich et al., 2010). Though there is consensus that CRT harms the brain, rodent models show a lack of reliability in producing robust cognitive impairment (Raber et al., 2004; Rola et al., 2004; Villasana et al., 2010). However, the standard behavioral tasks used typically rely on vision and thus may not be the most sensitive in detecting CRT impairment in the rodent. In this study, we used a recognition memory task that relies on olfaction to assess CRT-induced cognitive impairment because olfaction is an ethologically relevant sensory modality in the rodent. Additionally, neurogenesis in the subventricular zone (SVZ) and rostral migratory stream (RMS), a region through which new neurons travel to the olfactory bulb (Lledo et al., 2008), correlates with odor recognition memory (Lazarini et al., 2009; Gil-Perotin et al., 2011). Specifically, long-term recognition memory using the NOdorR task has been associated with greater levels of SVZ neurogenesis (Gil-Perotin et al., 2011). Previous work has demonstrated that SVZ to RMS neurogenesis is severely reduced by CRT (Hellström et al., 2009; Lazarini et al., 2009; Balentova et al., 2014). Because we (Rodgers et al., 2016) and others (Raber et al., 2004; Rola et al., 2004) have found reduced hippocampal neurogenesis months after cranial irradiation, DCX was used in this study to demonstrate a similar late effect in a neurogenic region (SVZ to RMS) associated specifically with the NOdorR task (Gil-Perotin et al., 2011). We reasoned that recognition memory of a social odor would be impaired 3 months post-CRT, thus providing a dependable means by which to detect CRT-induced cognitive impairment. We also included female mice because sex comparisons remain largely unexplored to date, even though there are reports of sex differences in pediatric survivors of CRT. In animals, there are only a few reported sex differences in behavioral outcomes after CRT which have generally indicated greater cognitive impairment in female rodents (Yazlovitskaya et al., 2006; Roughton et al., 2012) though this is influenced by dose of radiation (Villasana et al., 2010). We also used MRI after behavioral testing to evaluate CRT-induced volumetric and DTI changes in the brain. White matter pathology is typically associated with CRT-induced cognitive deficits in humans (Gragert and Ris, 2011), and MRI and DTI studies have shown that adult survivors of CRT have reduced cerebral volume (Armstrong et al., 2013) and white matter damage (Brinkman et al., 2012) via changes in FA and RD. Finally, histological assessment was performed to evaluate neurogenesis impairment along the RMS, an impairment associated with NOdorR (Gil-Perotin et al., 2011).

Therefore, the goals of this study were to determine acute and long-term effects of CRT on olfactory ability and memory in a mouse model as well as to quantify CRT-induced brain volume loss, white matter damage, and qualitative cellular changes in the RMS. In addition, these were examined in both sexes as female animal models have been largely unexplored. We hypothesized that CRT would (1) decrease RMS neurogenesis, reduce volume of brain regions important for olfactory memory, and cause white matter damage detectable via changes in FA or RD, and (2) that these would be associated with long-term olfactory memory impairment. We also hypothesized that these effects would be more severe in females.

## Materials and Methods

### Animals

Male (*n* = 24) and female (*n* = 36) C57BL/6J mice from Jackson Laboratory were housed in same-sex groups in a temperature-controlled room (22°C) and maintained on a 14/10-h light/dark cycle (lights on 6:00 a.m.–8:00 p.m.). All mice had *ad libitum* access to food and water. This study was carried out in accordance with the recommendations in the Guide for the Care and Use of Laboratory Animals of the National Institutes of Health. The protocol was approved by the Institutional Animal Care and Use Committee (IACUC) at Baylor College of Medicine and the IACUC at the University of Houston. Body weights were measured weekly and teeth trimmed if necessary (*n* = 1) under isoflurane anesthesia (100% oxygen with 2–3% isoflurane). Body lengths, the length from nose tip to base of tail, were measured the week prior to euthanasia.

### Cranial Irradiation

Males and females were randomly assigned to cranial irradiation (CRT) or control (SHAM) groups. CRT animals received whole-brain irradiation under isoflurane anesthesia while SHAM received only isoflurane. A lead shield was used to protect the tip of the snout to the backs of the eyes (approximately 7.0 bregma) to shield olfactory bulbs (located between the eyes) from irradiation. Another shield covered the rest of the body, leaving the whole brain exposed to a single dose of 5 Grays of X rays at a rate of approximately 110 cGy/minute using the RadSource 2000 with an energy beam of 160 kVp/25 mA. When deciding on this dose, we wanted to minimize unnecessary damage to the young mice and at the same time deliver enough damage that would allow us to investigate CRT effects on NOdorR task and its association with olfactory neurogenesis. Therefore, we chose this dose based on the reported observation by Rola et al. (2004) of a dose-dependent drop in cell proliferation that bottomed out starting from 5 Gy. Mice were irradiated at 4 weeks of age to model pre-adolescent human patients. This age shares two important similarities with the human population: (1) lack of sexual maturation (Silver, 1995; Goldman et al., 2007) and (2) incomplete brain development (Fu et al., 2013; Juraska et al., 2013). Specifically, prior to adolescence, regions within the amygdala, hypothalamic regions, the medial prefrontal cortex, and the visual cortex are not fully developed (Juraska et al., 2013).

### Habituation–Dishabituation

Acute (1 week post-CRT) and long-term (3 months post-CRT) effects on olfactory ability/discrimination were tested using the habituation–dishabituation test according to the protocol by Yang and Crawley (2009) with minor modifications. This task determined whether mice could smell by measuring sniffing times after repeated presentations of distinct odorants with short inter-trial intervals (ITI). Each odorant was presented for 90 s for three consecutive trials with an ITI of no more than 1 min. Non-social odorants were used in this task to ensure that mice could smell non-salient odorants without the involvement of the social chemosignals, or pheromones. The following odorants were used: almond, orange, lemon, and mint extract (McCormick, Hunt Valley) in 1:75 dilutions in water. Sniffing times for all trials were recorded using ODLog version 2.7.2 for Windows (Macropod).

### Novel Odor Recognition (NOdorR)

**FIGURE 1:**
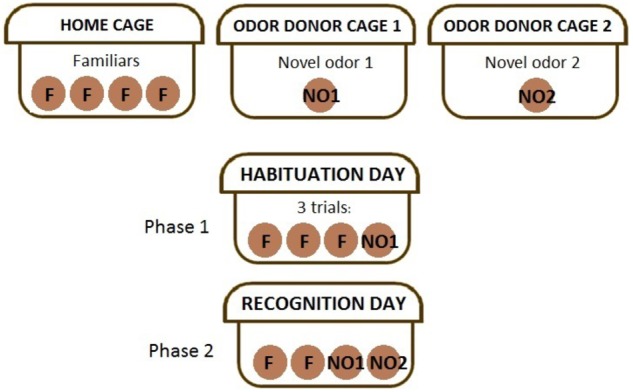
The experimental method of the novel odor recognition task. The top row describes the beads placed in cages according to their use. Habituation day or phase 1 of the task requires mice to sniff the first social odor, NO1, along with familiar (self-odor) beads in three trials. Recognition day or phase 2 of the task requires mice to sniff familiars, NO1, and NO2, a completely novel social odor.

The NOdorR task (Spinetta et al., 2008) was used to assess acute and long-term effects of CRT on olfactory ability/discrimination and recognition memory by presenting animals with ethologically relevant social odors. Processing of social odors involves both the vomeronasal and main olfactory systems (Richter et al., 2005; Dhungel et al., 2011). Social odors comprise a complex pattern of molecules that constitute an “odor signature” (Brennan and Kendrick, 2006). Accordingly, bedding was not changed for approximately 4–6 days prior to the experiment and 12 mm wooden beads (Woodworks Ltd.; Texas) were placed in (a) odor donor cages containing multiple C57Bl/6 mice of the opposite sex and (b) in the cages of subject mice for familiar odor beads (F1–F3) the day before the task began. Therefore, each mouse received ethologically relevant social odors via olfactory stimuli of the opposite sex, where each bead contained odors from multiple mice in cages that went unchanged for several days. Testing occurred over two days as follows (**Figure 1**): (1) Habituation day: F1–F3 were lined up at the front of the cage along with a fourth bead that had been left overnight in an odor donor cage (NO1), for three 1-min trials; (2) Recognition day: 24 h later, F1, F2, NO1, and a second novel odor bead (NO2; left overnight in a second cage of odor donor mice) were lined up at the front of the cage for a single 1-min trial. ODLog software was used to record time spent sniffing individual beads. Animals that did not explore both NO1 and NO2 were removed from data analysis (*n* = 1 male CRT at 1 week post-CRT and *n* = 1 female SHAM at 3 months post-CRT). A memory score was calculated for each group, [NO2/(NO1+NO2)] ^∗^100, where a score significantly above 50% demonstrates recognition memory of NO1 (Karlsson et al., 2015).

### Estrous Cycle Monitoring

The estrous cycle was monitored daily via vaginal smears for 4–5 days during the week of behavioral testing to determine stage of estrus, with the exception of the first testing period (1 week post-CRT) as 5-weeks-old mice are not sexually mature and do not have a regular estrous cycle at this age (Goldman et al., 2007). Vaginal smears were obtained at 4–6 p.m. after behavioral testing to reduce interference with behavior because vaginal stimulation can modify olfactory function (Larrazolo-López et al., 2008). Samples were taken by lifting the mouse by the base of the tail and placing it against the wall of the cage. According to previous methods (Caligioni, 2009), a “wet smear” was obtained by gently flushing the vaginal opening with less than 0.25 mL of phosphate buffered saline (PBS) and the final flush collected at the pipette tip placed on a glass slide. To determine stage of estrous (proestrus, estrus, metestrus, and diestrus), unstained samples were examined under a light microscope at 10× magnification with low illumination. For uniformity, male mice were handled similarly: instead of smears, light pressure was applied to the ano-genital area using a pipette tip.

### MRI Assessment of Volume and DTI

Animals were euthanized and perfused at 3 months post-CRT after the final behavioral task. At time of euthanasia, mice received an overdose of isoflurane anesthetic and were perfused transcardially with PBS followed by 4% paraformaldehyde (PFA) in phosphate buffer (pH 7.4) until body was stiff. Brains were prepared as described previously (Tyszka et al., 2006). Mouse brains were DTI scanned using 20 distinct gradient directions on a Bruker BioSpec MRI scanner (Bruker, MA) and tractography information generated. All details of computational processing using ROIEditor, DTI studio (Jiang et al., 2006), template maps from Duke Center for *In vivo* Microscopy (Calabrese et al., 2013), DiffeoMap (Zhang et al., 2009), AIR algorithm (Woods et al., 1998), and LDDMM (Ceritoglu et al., 2009) were presented previously (Sahnoune et al., 2018). Briefly, the diffusion-weighted maps were used to generate a brain mask using the skull strip function encoded in ROIEditor ^1^ followed by manual editing of the mask. DTI computations were performed using DTI studio^1^ to generate various volume maps: FA, relative anisotropy (RA), RD, anatomical (unweighted; B0), and color. Masked tensors were used to carry out fiber tracking with DTIstudio.

### Immunohistochemistry

After DTI imaging was completed, brains were transferred and stored in 70% ethanol solution until sectioning and paraffin embedding. Four sagittal sections were taken at lateral 0.84 and 1.08 mm (as defined in Franklin and Paxinos, 2008). Doublecortin (DCX) staining was used to label new, immature neurons (Francis et al., 1999) in the lateral ventricle (containing SVZ) and RMS regions for the purpose of qualitatively demonstrating a decrease in neurogenesis in the irradiated brains. Sections were first treated with 10mM citric acid for 10 min at 95°C for antigen retrieval. Sections were blocked for 30 min in 10% normal goat serum/0.2% Triton X in PBS. Then, sections were incubated with rabbit anti-DCX (1:400 dilution; Abcam; Cambridge, MA, United States) for approximately 24 h at 4°C followed by incubation in biotin-labeled secondary (goat; 1:200) for 2 h at room temperature. Next, sections were treated with the avidin–biotin complex (ABC, Vector Labs, Burlingame, CA, United States) for 1 h. Sections were then treated with diaminobenzidine (DAB kit, Vector Labs, Burlingame, CA, United States) for 2 min. Finally, sections were slide-mounted and counterstained with hematoxylin. Microscope images were captured using Leica Application Suite at 10X magnification under constant light intensity and imported into ImageJ (N.I.H., United States).

### Statistical Analysis

Body weight and olfactory discrimination data were analyzed by mixed-design ANOVAs with CRT and Sex as between-subjects variables and Age (for body weight data) or Trial (for habituation–dishabituation and phase 1 of NOdorR data) as within-subjects variables. In addition, because the habituation–dishabituation task requires the use of multiple odors, paired *t*-tests were used to analyze the “dishabituation” part of the task, the difference between the final trial of an odor and the first trial of a new odor, to determine if there was a significant change in exploration after a new odor was introduced. Final body lengths, volumetric data, DTI data, and memory scores between groups were analyzed by 2 × 2 (CRT × Sex) ANOVAs. Analysis of olfactory recognition memory performance (whether a group was able to perform this task) was carried out by comparing (1) NO1 versus NO2 using paired *t*-tests and (2) individual group memory scores against a control score of 50% using independent *t*-tests. Statistical analyses were performed using IBM SPSS for Windows (Version 23.0) and GraphPad Prism 6 software (GraphPad, La Jolla, CA, United States). Statistical significance was set at *p* < 0.05.

## Results

### The Effect of CRT on Body Weight and Length

**FIGURE 2:**
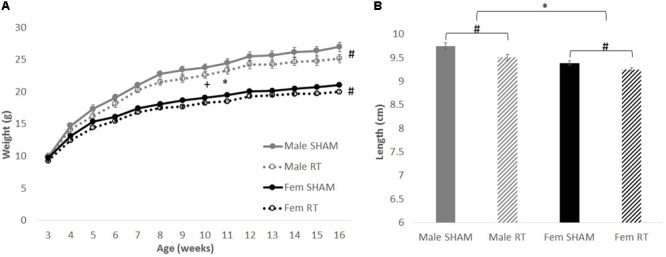
The effect of radiation on body weight and length. **(A)** Weights over time up to approximately 3 months post-CRT. Male mice weighed more than females throughout late adolescence and adulthood. Irradiated mice consistently weighed less than their sex-matched control counterparts. **(B)** Body lengths at approximately 3 months post-CRT. Males were larger than females and irradiated were smaller than their sex-matched controls. All data are presented ± SEM. ^∗^*p* < 0.05 indicates sex difference. ^#^*p* < 0.05 indicates main effect of radiation. ^+^*p* < 0.001 indicates effect of age.

Overall, body weights and lengths of CRT animals were smaller than their sex-matched controls (**Figure 2A**). For body weights, Mauchly’s test showed that the assumption of sphericity had been violated for Age, χ^2^(90) = 793.23, *p* < 0.001. Therefore, degrees of freedom were corrected using Greenhouse–Geisser estimates of sphericity (𝜀 = 0.17). There were significant effects of age [*F*(2.16,121.11) = 1559.48, *p* < 0.001] and age × sex [*F*(2.16,121.11) = 65.51, *p* < 0.001], but not CRT × age [*F*(2.16,121.11) = 2.03, *p* > 0.05] or CRT × sex × age [*F*(2.16,121.11) = 0.63, *p* > 0.05]. There were also significant effects of CRT [*F*(1,56) = 6.99, *p* = 0.01] and sex [*F*(1,56) = 118.42, *p* < 0.001] but not CRT × sex [*F*(1,56) = 0.25, *p* > 0.05]. Analysis of body lengths at approximately 3 months post-CRT (**Figure 2B**) resulted in significant effects of CRT [*F*(1,56) = 13.06, *p =* 0.001] and Sex [*F*(1,56) = 34.22, *p* < 0.001] but no interaction [*F*(1,56) = 0.83, *p* > 0.05].

### Acute Effects of CRT: Olfactory Capability and Discrimination

**FIGURE 3:**
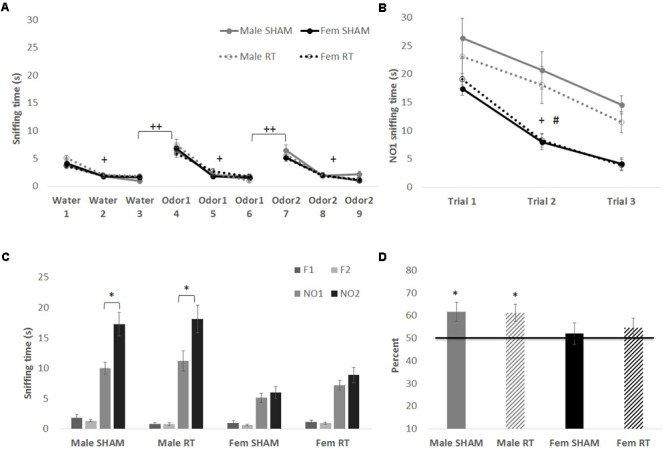
Odor tasks at 1 week post-CRT. **(A)** Habituation–dishabituation task with non-social odors. All mice showed a similar decline in investigation of an odor with repeated trials (habituation), and an increase with the introduction of a novel odor (dishabituation). **(B)** The first phase of the novel odor recognition task, exploration of a novel social odor. All mice showed a similar decline in investigation of an odor with repeated trials, with males overall spending more time investigating odors. **(C)** The second phase of the novel odor recognition task, the recognition test at 1 week post-CRT. Males, regardless of radiation, showed odor recognition memory via significantly greater exploration of the most novel odor (NO2) in comparison to NO1. **(D)** This is shown again via the memory score, NO2/(NO1+NO2) with scores significantly above chance (50%). All data are presented ± SEM. ^#^*p* < 0.001 indicates sex difference. ^+^*p* < 0.001 for habituation to odor for all groups. ^++^*p* < 0.001 for dishabituation to an odor for all groups. ^∗^*p* < 0.05 indicates NO2 > NO1 or memory score significantly above 50%.

All groups had intact olfactory capability, based on the habituation–dishabituation task at 1 week post-CRT (**Figure 3A**). Mauchly’s test indicated that the assumption of sphericity had been violated for Trial for each odor [water: χ^2^(2) = 12.67, *p* < 0.01; first novel odorant: χ^2^(2) = 42.90, *p* < 0.001; second novel odorant: χ^2^(2) = 26.46, *p* < 0.01]. Therefore, degrees of freedom were corrected using Greenhouse–Geisser estimates of sphericity (𝜀 = 0.83, 0.65, and 0.72, respectively). Habituation to an odor (indicated by a decrease in exploration) was shown by a significant effect of trial on sniffing time for water [*F*(1.66,92.89) = 92.37, *p* < 0.001] but no interactions [trial × sex: *F*(1.66,92.89) = 2.12 and trial × CRT: *F*(1.66,92.89) = 0.21; *p* > 0.05]. The effects of CRT [*F*(1,56) = 1.58], sex [*F*(1,56) = 0.23], and their interaction [*F*(1,56) = 2.54] were not significant [*p* > 0.05]. Similarly, habituation was observed for the first novel odorant [trial: *F*(1.30,72.65) = 137.23, *p* < 0.001] with no significant interactions [trial × sex: *F*(1.30,72.65 = 0.93 and trial × CRT: *F*(1.30,72.65) = 0.91; *p* > 0.05] or between-subjects effects [sex: *F*(1,56) = 0.05, CRT: *F*(1,56) = 0.05, and CRT × sex: *F*(1,56) = 0.04; *p* > 0.05]. The same was found for the second novel odorant [trial: *F*(1.45,81.05) = 109.04, *p* < 0.001; trial × sex: *F*(1.45,81.05) = 1.24, trial × CRT: *F*(1.45,81.05) = 0.24, sex: *F*(1,56) = 1.56, CRT: *F*(1,56) = 0.52, and CRT × sex: *F*(1,56) = 0.65, *p* > 0.05]. As expected, animals demonstrated dishabituation (an increase in exploration) with the introduction of a novel odorant. Sniffing time was significantly greater for trial 4 than trial 3 [male SHAM: *t*(11) = 7.98, male CRT: *t*(11) = 6.52, female SHAM: *t*(17) = 6.70, and female CRT: *t*(17) = 5.88; *p* < 0.001], and greater for trial 7 than trial 6 [male SHAM: *t*(11) = 4.37, *p* = 0.001; male CRT: *t*(11) = 6.22, female SHAM: *t*(17) = 5.64, and female CRT: *t*(17) = 5.05; *p* < 0.001]. In agreement with the habituation–dishabituation results, mice showed habituation to a novel social odor (NO1) in the first phase of the NOdorR (**Figure 3B**; sniffing times of familiar odors are not shown due to exceptionally low exploration levels from all groups, 0–2 s). Mauchly’s test indicated that the assumption of sphericity had been violated for Trial for NO1 [χ^2^(2) = 25.55, *p* < 0.001] and therefore degrees of freedom were corrected using Greenhouse–Geisser estimates of sphericity (𝜀 = 0.73) [trial: *F*(1.46,81.66) = 42.54, *p* < 0.001]. There were no significant interactions [trial × sex: *F*(1.46,81.66) = 1.43 and trial × CRT: *F*(1.46,81.66) = 0.05; *p* > 0.05]. For habituation to NO1, there was a significant main effect of sex [*F*(1,56) = 68.10, *p* < 0.001] but no effect of CRT [*F*(1,56) = 1.22, *p* > 0.05] and no interaction [*F*(1,56) = 2.74, *p* > 0.05]. Overall, sniffing times for NO1 were higher for males than females.

### Acute Effects of CRT: Olfactory Recognition Memory

In the second and final phase of the NOdorR test, mice were tested for odor recognition memory with a 24-h retention interval (**Figures 3C,D**). Male SHAM [*t*(11) = 2.88, *p* < 0.05] and male CRT [*t*(10) = 2.52, *p* < 0.05] investigated the most novel odor (NO2) more than the previously exposed odor (NO1), but female SHAM [*t*(17) = 0.81, *p* > 0.05] and female CRT [*t*(17) = 1.23, *p >* 0.05] did not. Similarly, individual memory scores for both male SHAM [*t*(11) = 2.76, *p <* 0.05] and male CRT [*t*(10) = 3.00, *p <* 0.05] were significantly above 50%, indicating recognition of NO1. This was not the case in either of the female groups [female SHAM, *t*(16) = 0.43, *p* > 0.05; female CRT, *t*(16) = 1.06, *p* > 0.05]. A two-way ANOVA (CRT × sex) on memory scores demonstrated a lack of significant results [CRT: *F*(1,57) = 0.06, sex: *F*(1,57) = 3.21, and CRT × sex: *F*(1,57) = 0.10; *p* > 0.05].

### Long-Term Effects of CRT: Olfactory Capability and Discrimination

**FIGURE 4:**
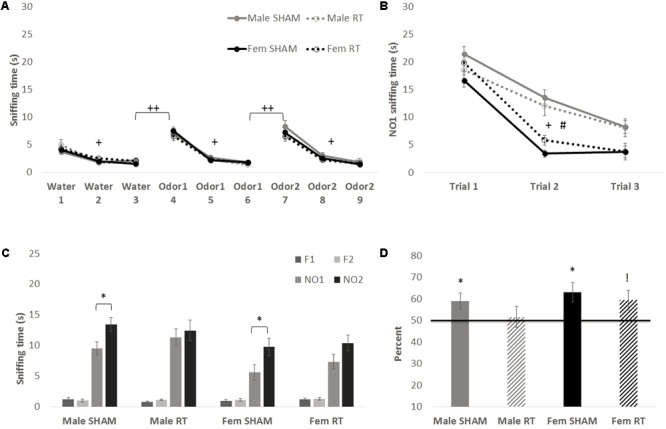
Odor tasks at 3 months post-CRT. **(A)** Habituation–dishabituation task with non-social odors. All mice showed a similar decline in investigation of an odor with repeated trials (habituation), and an increase with the introduction of a novel odor (dishabituation). **(B)** The first phase of the novel odor recognition task, exploration of a novel social odor. All mice showed a similar decline in investigation of an odor with repeated trials, with males overall displaying greater investigation of the odor. **(C)** The second phase of the novel odor recognition task, the recognition test at 3 months post-CRT. Non-irradiated males and females showed odor recognition memory via significantly greater exploration of the most novel odor (NO2) in comparison to NO1. **(D)** This is shown again via the memory score, NO2/(NO1+NO2) with significance indicating a score above chance or 50%, though irradiated females indicate a trend toward significance. All data are presented ± SEM. ^#^*p* < 0.001 indicates sex difference. ^+^*p* < 0.001 for habituation to odor for all groups. ^++^*p* < 0.001 for dishabituation to an odor for all groups. ^∗^*p* < 0.05 indicates NO2 > NO1 or memory score significantly above 50%. ^!^*p* = 0.05 indicates trend above 50%.

At 3 months post-CRT, results of the habituation–dishabituation task (**Figure 4A**) were similar to those obtained 1 week post-CRT. Mauchly’s test indicated that the assumption of sphericity had been violated for Trial for each non-social odor [water: χ^2^(2) = 22.68, first novel odorant: χ^2^(2) = 44.87, and second novel odorant: χ^2^(2) = 32.41; *p* < .001]. Therefore, degrees of freedom were corrected using Greenhouse–Geisser estimates of sphericity (𝜀 = 0.75, 0.64, and 0.69, respectively). Animals habituated to the first non-social odorant (water) as shown by a significant effect of Trial [*F*(1.50,83.71) = 61.32, *p* < 0.001] with no interactions [trial × sex: *F*(1.50,83.71) = 1.18 and trial × CRT: *F*(1.50,83.71) = 1.00; *p* > 0.05] and no significant between-groups effects [sex: *F*(1,56) = 0.02, CRT: *F*(1,56) = 1.58, and CRT × sex: *F*(1,56) = 0.06; *p* > 0.05]. The same was found for the first novel odorant [trial: *F*(1.28,71.90) = 156.03, *p* < 0.001; trial × sex: *F*(1.28,71.90) = 0.12, trial × CRT: *F*(1.28,71.90) = 0.05, sex: *F*(1,56) = 0.004, CRT: *F*(1,56) = 0.35, and CRT × sex: *F*(1,56) = 0.03; *p* > 0.05] and the final novel odorant [trial: *F*(1.38,77.50) = 174.40, *p* < 0.001; trial × sex: *F*(1.38,77.50) = 0.08, trial × CRT: *F*(1.38,77.50) = 2.26, sex: *F*(1,56) = 0.87, CRT: *F*(1,56) = 1.42, CRT × sex: *F*(1,56) = 0.31; *p* > 0.05]. Moreover, animals showed an increase in exploration from trial 3 to trial 4 [male SHAM: *t*(11) = 5.43, male CRT: *t*(11) = 7.31, female SHAM: *t*(17) = 9.07, and female CRT: *t*(17) = 5.71; *p* < 0.001] and from trial 6 to 7 [male SHAM: *t*(11) = 6.29, male CRT: *t*(11) = 5.40, female SHAM: *t*(17) = 6.25, and female CRT: *t*(17) = 7.42; *p* < 0.001].

In the first phase of the NOdorR task (**Figure 4B**), all mice demonstrated habituation to NO1 [trial: *F*(2,110) = 111.33, *p* < 0.001] with a significant trial × sex [*F*(2,110) = 6.09, *p* < 0.01] but not trial × CRT [*F*(2,110) = 0.04, *p* > 0.05] effect. Between groups, there was also a main effect of sex [*F*(1,55) = 31.90, *p* < 0.001] where males investigated this social odor more than females, but no main effect of CRT [*F*(1,55) = 0.06, *p* > 0.05] or interaction [*F*(1,55) = 4.03, *p =* 0.05].

### Long-Term Effects of CRT: Olfactory Recognition Memory

Three months post-CRT, mice were again tested on odor recognition memory 24 h after their first NO1 exposure (**Figures 4C,D**). Male SHAM [*t*(11) = 2.60, *p* < 0.05] and female SHAM [*t*(16) = 2.47, *p* < 0.05] investigated NO2 significantly more than NO1, whereas male CRT [*t*(11) = 0.50, *p* > 0.05] and female CRT [*t*(17) = 1.70, *p >* 0.05] did not. Similarly, individual memory scores for male SHAM [*t*(11) = 2.46, *p* < 0.05] and female SHAM [*t*(16) = 2.99, *p* < 0.05] were significantly above 50%, and it should be noted that female CRT was at the level of significance [*t*(17) = 2.06, *p* = 0.05]. In contrast, male CRT explored both odors equally [*t*(11) = 0.32, *p* > 0.05], indicating lack of recognition of NO1 in male CRT. A two-way ANOVA (CRT × sex) on memory scores was not significant [CRT: *F*(1,59) = 1.49, sex: *F*(1,59) = 1.69, and interaction: *F*(1,59) = 0.17; *p* > 0.05].

**FIGURE 5:**
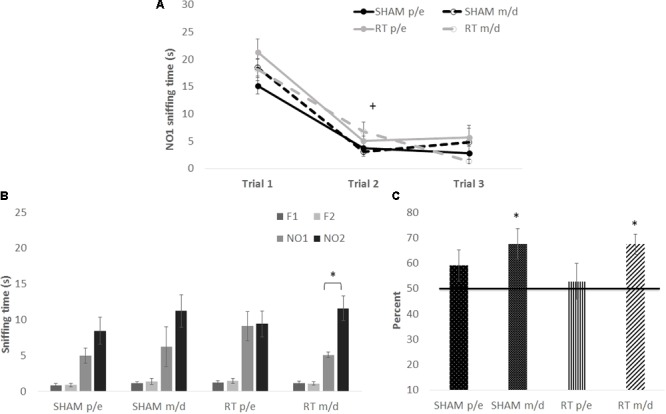
The habituation and recognition task at 3 months post-CRT, divided up by stage of estrus among female mice. “p/e” indicates proestrus/estrus and “m/d” indicates metestrus/diestrus stages of the cycle. **(A)** All females, regardless of stage of estrus or CRT, had similar habituation or decline in exploration of the first social odor exposure. **(B)** Irradiated females showed odor recognition memory via significantly greater exploration of the most novel odor (NO2) in comparison to NO1. **(C)** Both non-irradiated females and irradiated females in m/d stages of the cycle show recognition memory of NO1. All data are presented ± SEM. + indicates *p* < 0.001 for habituation to odor for all groups. ^∗^*p* < 0.05 indicates NO2 > NO1 or memory score significantly above 50%.

However, it has been shown that the estrous cycle influences the memory consolidation phase of the social recognition memory test (Sanchez-Andrade and Kendrick, 2011), a task where odor is the primary cue. Therefore, the stage of estrus was examined on the day of NO1 habituation, the memory-consolidation equivalent of the NOdorR task, and female recognition memory scores were divided into “proestrus/estrus” and “metestrus/diestrus” (**Figure 5**). Habituation to NO1 [**Figure 5A**; trial: *F*(2,64) = 94.77, *p* < 0.001] was similar among all females as there were no significant group differences [CRT: *F*(1,32) = 3.05 and estrous: *F*(1,32) = 0.05; *p* > 0.05] or interactions [trial × estrous: *F*(2,64) = 0.29, trial × CRT: *F*(2,64) = 1.13, and CRT × estrous: *F*(1,32) = 3.23; *p* > 0.05]. Recognition memory, however, varied by stage of estrus on NO1 habituation (consolidation) day. When comparing NO1 versus NO2 (**Figure 5B**), only female CRT in metestrus/diestrus reached significance [*t*(7) = 3.90, *p* < 0.05]. In terms of memory scores (**Figure 5C**), both female SHAM [*t*(7) = 2.88, *p* < 0.05] and female CRT [*t*(7) = 4.48, *p* < 0.05] in metestrus/diestrus were significantly above 50%. This was not found in female SHAM and CRT in proestrus/estrus [*t*(8) = 1.45, *p >* 0.05; *t*(9) = 0.40, *p >* 0.05, respectively]. A two-way ANOVA (CRT × estrous) on memory scores was not significant [CRT: *F*(1,31) = 0.26, estrous: *F*(1,31) = 3.50, and interaction: *F*(1,31) = 0.26; *p* > 0.05].

### Neural Effects of CRT

**FIGURE 6:**
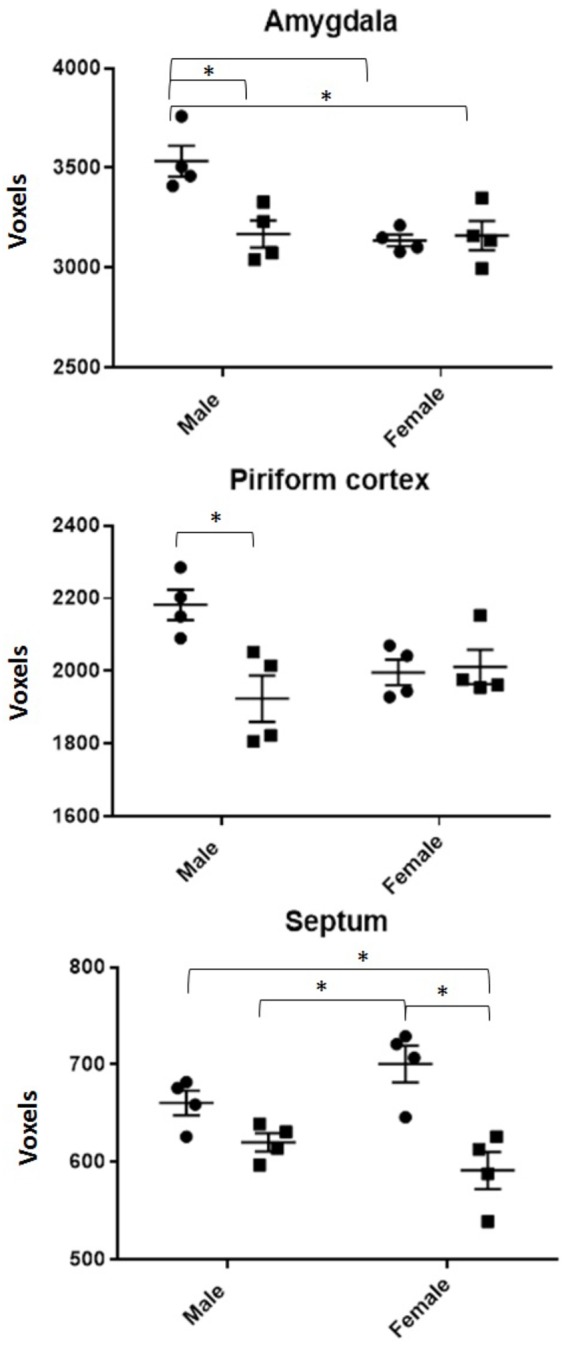
All volumes of regions demonstrating a radiation by sex interaction effect. SHAM is denoted by circles, CRT by squares. ^∗^ denotes a significant pairwise comparison. Males generally show CRT deficits in both the piriform cortex and amygdala but not the septum.

**FIGURE 7:**
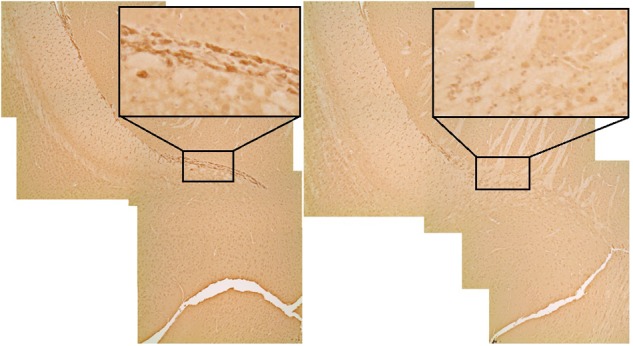
DCX (immature neurons) staining in the rostral migratory stream. The irradiated animal on the right shows a qualitative reduction (black box) in olfactory neurogenesis in comparison to the sham on the left.

DTI analyses at 3 months post-CRT demonstrated a significant reduction in volumes of many brain regions, including several involved in social odor exploration and recognition memory: amygdala, fimbria, hippocampus, hypothalamus, piriform cortex, and neocortex (**Table 1**). The olfactory bulb, which was shielded during irradiation, was not reduced in volume [CRT: *F*(1,16) = 0.98, *p* > 0.05]. Three regions showed a significant CRT × sex effect (**Figure 6**): the amygdala [*F*(1,16) = 9.06, *p* < 0.05], the piriform cortex [*F*(1,16) = 8.07, *p* < 0.05], and the septum [*F*(1,16) = 4.89, *p* < 0.05]. There were also several significant sex effects in volume (see **Table 1**). There were no effects of irradiation on FA or RD, with the exception of fornix RD [*F*(1,16) = 5.07, *p* < 0.05; Supplementary Table S2]. A few sex differences among FA and RD values were also observed (Supplementary Tables S1, S2, respectively). Additionally, a noticeable reduction of DCX (new, migrating neurons) staining was observed in CRT in comparison to SHAM as observed in the RMS (**Figure 7**).

**Table 1:** Volumes of all brain structures.

Brain region	Sex			Radiation		Sex^∗^radiation	
	*F*-value	*p*		*F*-value	*p*	*F*-value	*p*
Accumbens nucleus	8.52	**0.013**	M > F	7.88	**0.016**	1.392	0.261
Amygdala	9.849	**0.009**		6.905	**0.022**	9.064	**0.011**
Anterior commissure	0.014	0.909		0.701	0.419	0.01	0.921
Caudate putamen	0.042	0.841		15.617	**0.002**	0.296	0.597
Cerebellum	8.987	**0.011**	M > F	14.483	**0.003**	0.004	0.953
Cingulum	25.075	**<0.001**	F > M	6.598	**0.025**	2.616	0.132
Claustrum	4.145	0.064		5.652	**0.035**	1.167	0.301
Corpus callosum and external capsule	19.742	**0.001**	F > M	12.079	**0.005**	0.311	0.587
Dorsal and ventral endopiriform nucleus	9.514	**0.009**	F > M	0.788	0.392	0.229	0.641
Fasiculus retroflexus	0.041	0.844		1.629	0.226	0.762	0.4
Fimbria	26.521	**<0.001**	F > M	10.457	**0.007**	1.442	0.253
Fornix	5.869	**0.032**	M > F	2.041	0.179	0.062	0.808
Hippocampus	1.348	0.268		24.772	**<0.001**	0.001	0.978
Hypothalamus	0.069	0.798		12.565	**0.004**	0.003	0.959
Inferior colliculus	1.448	0.252		10.232	**0.008**	0.688	0.423
Internal capsule	6.296	**0.027**	F > M	16.959	**0.001**	1.732	0.213
Lateral globus pallidus	0.044	0.837		3.495	**0.086**	0.018	0.895
Mammillothalamic tract	0.13	0.725		5.966	**0.031**	3.542	0.084
Neocortex	0.144	0.711		15.0	**0.002**	0.225	0.643
Nosebulb	0.275	0.610		0.981	0.341	0.244	0.63
Optic tract	0.054	0.82		2.398	0.147	0.784	0.393
Periaqueductal gray	0.615	0.448		4.441	0.057	1.808	0.204
Piriform cortex	1.051	0.326		6.369	**0.027**	8.072	**0.015**
Septum	0.131	0.724		23.184	**<0.001**	4.886	**0.047**
Stria medularis	2.575	0.135		0.543	0.476	0.224	0.644
Stria terminalis	2.426	0.145		2.362	0.15	1.129	0.309
Superior colliculus	0.313	0.586		6.375	**0.027**	0.027	0.873
Thalamus	0.199	0.663		13.743	**0.003**	0.648	0.436
Ventricles	5.537	0.037	M > F	47.781	**<0.001**	2.021	0.181
Whole brain	8.192	0.014	M > F	16.777	**0.001**	0.072	0.793

*Radiation reduced several brain regions in volume, including structures involved in olfactory processing and social odor recognition memory. All significant *p*-values are shown in bold. Sex differences are further denoted by M and F for male and female, respectively.*

## Discussion

The goal of this study was to determine whether CRT during development induces olfactory memory impairment in male and female mice. While some have previously reported cognitive impairment with visual tasks, we are not aware of any study on the effect of cranial irradiation on olfactory memory using the NOdorR task in both males and females. We found that irradiated males demonstrated odor recognition memory impairment in adulthood (3 months post-CRT) but no radiation-induced impairments were observed 1 week post-CRT in either sex. Impairment of odor recognition memory was not due to a lack of ability to smell, as all mice showed appropriate habituation to non-social and social odors. However, female performance was dependent on hormonal status, as only females in metestrus or diestrus showed odor recognition memory, regardless of CRT. Qualitative histological evaluation indicates a reduction in olfactory neurogenesis in the RMS. Brain MRI demonstrated general detrimental effects of irradiation, with specific regions showing interactive effects of CRT by sex, suggesting their involvement in social odor recognition memory.

### Sex Differences in Post-CRT Odor Recognition Memory

Both acute and long-term testing showed that all mice demonstrated habituation (decreasing exploration to repeated exposures of an odorant) and dishabituation (increased investigation of a new odorant) to non-social odors, indicating intact olfactory ability. In addition, all mice habituated to the first social odor (NO1), but in this case males explored the stimuli longer than females did, a result that has been observed previously with social stimuli (Karlsson et al., 2015). There were no effects of CRT on general exploration of any odors.

However, irradiated male mice demonstrated impaired social odor recognition memory in adulthood, despite all males having a similar habituation curve to NO1. No CRT-induced recognition memory impairment was observed in the acute testing period, and the late onset of cognitive impairment is consistent with the clinical literature (Ullrich and Embry, 2012). To our knowledge, only one other study has demonstrated long-term recognition memory impairment using the NOdorR task after whole-body irradiation (Mange et al., 2018) and their findings also confirm long-term cognitive impairment in males. Females, on the other hand, at first glance appear to show a similar CRT impairment in odor recognition memory, but further investigation showed that this depended on stage of estrus, with no effect due to irradiation. In females, odor recognition memory in adulthood was dependent on the stage of estrus and thus, presumably, circulating gonadal hormones: females that were in metestrus/diestrus during habituation (consolidation) of NO1 showed intact olfactory recognition memory whereas proestrus/estrus females did not. Though our findings are not consistent with a previous study on proestrus-dependent consolidation in social recognition memory (Sanchez-Andrade and Kendrick, 2011), a similar task to odor recognition that typically employs juvenile animals as stimuli, the inconsistency could be due to differences in methodology, particularly in the stimuli used. The present study employed adult, opposite sex odors, which could mean that sexual or reproductive behaviors played a role in our study. For example, Markham and Juraska (2007) found that female rats in proestrus and estrus demonstrated social recognition memory with female stimuli but not with male stimuli, which generally agrees with our findings. Additionally, estrous females prefer adult, dominant male odors over subordinate male odors (Mossman and Drickamer, 1996), a factor not controlled in the present study. However, differences in odor recognition were not due to prior exploration levels of NO1 as there were no differences between females during habituation to NO1.

In contrast to adulthood behavior, juvenile animals did not demonstrate any acute radiation-induced impairment at 1 week post-CRT. Instead, females, but not males, displayed a lack of odor recognition during adolescence, suggesting a possible sex difference in the development of murine behaviors toward social odors. At this time point (5 weeks of age), mice have not reached sexual maturity (Jackson Laboratory, 2009). Sexual maturation is affected by cranial irradiation as it can accelerate or inhibit puberty in a dose-dependent manner (Roth et al., 2000), and female mice have likely not acquired a regular estrous cycle (Goldman et al., 2007), a factor that proved to be tightly linked to odor recognition memory in adulthood. Hormone-induced postnatal development of female sexual behaviors occurs later in female mice in comparison to male-typical behaviors (Brock et al., 2011), and in females, specific reproductive behaviors are further influenced by progesterone (Dey et al., 2015) and its receptors (Desroziers et al., 2017), though the role that progesterone plays in the sexually maturing brain remains unclear. In the present study, females showed odor recognition memory only during the metestrus and diestrus estrous stages in adulthood, a period characterized by increasing progesterone (Wood et al., 2007; Aritonang et al., 2017), but the same was not found during early adolescence, a period of continuing sexual development.

### Imaging and Histological Assessment Demonstrate CRT Damage to the Brain

Most irradiated brain regions were reduced in volume in adulthood, including the hippocampus, amygdala, neocortex, hypothalamus, and piriform cortex, demonstrating widespread effects of CRT. As expected, we also observed a qualitative indication of reduction of new neurons in the RMS, suggesting impaired olfactory neurogenesis after irradiation, a result that has been previously shown (Hellström et al., 2009; Lazarini et al., 2009; Balentova et al., 2014). Reduced olfactory neurogenesis has been associated with impairment in the NOdorR task (Gil-Perotin et al., 2011). Moreover, similar to our work, others have shown intact olfactory ability yet long-term olfactory memory impairment via odor-association task (Lazarini et al., 2009), as well as emotional memory deficit in an olfactory fear conditioning test (Valley et al., 2009). However, in contrast to our work, these studies not only used less ethological (non-social) odors, but only the SVZ and/or RMS was irradiated instead of the whole brain, a more clinically relevant method for pediatric radiotherapy. Additionally, Lazarini et al. (2009) used an olfactory associative task that required a period of training which does not test basic recognition memory and is a task that more specifically involves frontal olfactory cortices (Roullet, 2005). Furthermore, other forms of neurogenesis ablation in the SVZ also demonstrate intact ability to smell or olfactory discrimination (Imayoshi et al., 2008; Sakamoto et al., 2011), as we have shown in the present study.

Olfactory memory consolidation requires a distributed neuronal circuitry (Sanchez-Andrade et al., 2005), and therefore the multitude of volumetric reductions via MRI demonstrate broad damage to olfactory neural processing. Additionally, the olfactory bulbs, the first in line in odor processing and a region shielded during CRT, did not show a volume decrease which suggests a lack of damage in the olfactory bulbs. Similar to previous work, we found sex differences in volume of specific brain regions, including the larger whole brain volume, as well as larger amygdala in males, and a larger fimbria in females (Koshibu et al., 2004; Spring et al., 2007), which is similar to those reported in humans (Goldstein et al., 2001). Interestingly, three regions involved in olfactory processing and/or consolidation of a social odor memory – the piriform cortex, amygdala, and septum (Everts and Koolhaas, 1997; Ferguson et al., 2001; Richter et al., 2005; Sanchez-Andrade et al., 2005; de Castro, 2009; Martin, 2012) – showed an interaction between sex and CRT, which are of interest due to the results of the odor recognition task. The amygdala and piriform cortex were reduced in irradiated males but not females, a pattern similar to olfactory memory performance, suggesting that these regions were involved in radiation-induced impairment of odor recognition memory. While the piriform cortex, a large region of cortex in the rodent, is involved in the general processing of any type of odor (Tronel and Sara, 2002; Richter et al., 2005; Roullet, 2005), both the piriform cortex and amygdala have additionally been implicated in a social recognition memory task with a similar paradigm to our 24 h odor recognition task (Richter et al., 2005; Tanimizu et al., 2017), as well as integration of olfactory information and maintenance of social odor recognition memory (Brennan and Kendrick, 2006). The medial amygdala in particular has shown greater neuronal activation via female odors in comparison to male odors in male rodent subjects (Donato et al., 2010), a finding relevant to our results as we used opposite-sex odors. Furthermore, intact social recognition memory of a female requires oxytocin in the medial amygdala but not other brain regions (Ferguson et al., 2001). Finally, though the lateral septum originates from the medial amygdala, the volume of the septum was the only region with a sex by CRT interaction that did not match cognitive performance results: the male septum volume did not change while females showed a reduction by CRT. Vasopressin in the lateral septum has been highly associated with successful social recognition memory in male rats (Bluthe et al., 1990; Everts and Koolhaas, 1997), a region that contains greater vasopressin fibers in males compared to females (de Vries et al., 1981) and thus may be a structure robust against radiation damage in males. Together, this suggests that the septum is not involved in radiation-induced odor recognition impairment.

Additionally, there is the possibility that females are displaying a form of neuroprotection. Because females did not show the late effects induced by CRT that males demonstrated (odor recognition memory impairment and volume reduction in regions important for olfactory processing), this leaves the possibility that estrogen, particularly estradiol, may play a neuroprotective role (Suzuki et al., 2006) after radiation damage. For example, previous research has found that estradiol can be protective in the adult after perinatal asphyxia (Saraceno et al., 2018) and other forms of CNS damage, including an animal model of cerebrovascular stroke (Suzuki et al., 2006).

We did not observe any significant changes in FA or RD in the different brain regions following CRT or interactive effects between CRT and sex between the animals. These findings are similar to our results in rats when we did not find significant changes in FA but detected connectome differences due to CRT and exercise (Sahnoune et al., 2018). Similarly, others have also reported difficulty in measuring differences in FA in animal models of CRT (Wang et al., 2009).

### Body Changes After CRT Damage

Prior to examining long-term damage to the brain, we observed radiation-induced decreases in body lengths at 3 months post-CRT and weights over time. Our group has previously observed this in rats irradiated as juveniles (Rodgers et al., 2016; Sahnoune et al., 2018), with a similar consistent lower body weight in irradiated animals in comparison to shams. Further, the present study demonstrated sex differences in body weight with no interaction with CRT: adult male mice consistently weighed more than females.

### Limitations and Future Directions

While males demonstrated radiation-induced behavioral impairment, females instead primarily showed an effect of estrous cycle. Our findings suggest that the NOdorR task using social odors is not a suitable test for CRT-induced impairment in female mice. Future assessments in females may include behavioral tasks that are not hormone-dependent or are controlled for estrous cycling. Additionally, whereas within-group analyses (memory score at or above 50%) demonstrated impairment in irradiated males, between-groups analysis of memory scores demonstrated a lack of significance. That is, control mice (males in particular) explore the most novel odor only a little more than the previous social odor, likely due to the salience of these social odors. Therefore, the use of adult, opposite sex odors in our study may have limited our ability to observe robust radiation-induced memory impairment in both sexes. Greater habituation (additional trials) to the first odor exposure may be necessary to reveal robust differences between groups.

Imaging data primarily demonstrated volumetric differences. We did not observe any DTI changes due to CRT, whereas human survivors of CRT display white matter damage associated with cognitive impairment (Gragert and Ris, 2011). However, volume differences showed reduced overall brain tissue in CRT animals, particularly regions with sex-dependent effects that matched odor recognition impairment (amygdala and piriform cortex). Future research could be dedicated to investigating the roles of the amygdala and piriform cortex in social odor recognition among males and females. Special attention could be dedicated to the medial amygdala due to its role in social odor processing.

## Data Availability

The raw data supporting the conclusions of this manuscript will be made available by the authors, without undue reservation, to any qualified researcher.

## Author Contributions

EP, JLL, and MWG planned the experiments and prepared the manuscript. EP performed the experiments and processed the data. SR contributed to statistical analyses, experimental design, and editing of the manuscript. TI contributed to experiments. SP processed and analyzed the imaging data.

## Conflict of Interest Statement

The authors declare that the research was conducted in the absence of any commercial or financial relationships that could be construed as a potential conflict of interest.

## Footnotes

1 www.mristudio.org

## Abbreviations

CRT: cranial radiation therapy
DTI: diffusion tensor imaging
FA: fractional anisotropy
MRI: magnetic resonance imaging
NOdorR: novel odor recognition
RD: radial diffusivity
RMS: rostral migratory stream
