# Supplementary material for: Olfactory Memory Impairment Differs by Sex in a Rodent Model of Pediatric Radiotherapy
Source: Front Behav Neurosci. 2018 Aug 2;12:158. doi: 10.3389/fnbeh.2018.00158 (PMC6084003; doi:10.3389/fnbeh.2018.00158)
Supplement: Supplementary file 1 [file Table_1.pdf]

Supplementary Material

Olfactory Memory Impairment Differs by Sex in a Rodent Model of Pediatric Radiotherapy

Emma C. Perez[1,2*], Shaefali P. Rodgers[1], Taeko Inoune[2], Steen E. Pedersen[3,4], J. Leigh Leasure[1,5*], M. Waleed Gaber[2,3*]

*** Correspondence:** Dr. M. Waleed Gaber: gaber@bcm.edu

**Brain region (FA)**	**Sex**			**Radiation**		**Sex*Radiation**	
	**F value**	**p**		**F value**	**p**	**F value**	**p**
Accumbens nucleus	4.08	0.066		0.00	0.963	0.40	0.538
Amygdala	3.00	0.109		0.03	0.877	2.14	0.169
Anterior commissure	1.52	0.241		0.08	0.779	0.18	0.683
Caudate putamen	2.90	0.114		2.85	0.117	2.717	0.125
Cerebellum	0.56	0.47		2.88	0.115	1.24	0.288
Cingulum	1.42	0.256		0.01	0.943	0.22	0.648
Claustrum	3.13	0.102		0.02	0.880	2.55	0.136
Corpus callosum & external capsule	5.99	**0.031**	M>F	0.01	0.916	0.16	0.698
Dorsal & ventral endopiriform nucleus	1.18	0.298		0.08	0.785	2.73	0.124
Fasiculus retroflexus	3.57	0.083		0.49	0.497	0.26	0.617
Fimbria	1.73	0.213		0.14	0.717	0.15	0.71
Fornix	0.47	0.508		0.39	0.546	0.01	0.907
Hippocampus	2.55	0.136		0.82	0.382	0.01	0.945
Hypothalamus	2.56	0.135		0.84	0.379	1.96	0.186
Inferior colliculus	0.22	0.648		2.07	0.176	0.97	0.345
Internal capsule	2.82	0.119		0.07	0.798	0.11	0.743
Lateral globus pallidus	0.02	0.904		0.22	0.645	1.34	0.27
Mammillothalamic tract	0.04	0.841		0.01	0.942	0.32	0.58

Neocortex	0.74	0.408		0.37	0.553	0.15	0.702
Nosebulb	7.10	**0.021**	M>F	0.00	0.963	0.96	0.348
Optic tract	2.22	0.162		0.17	0.687	0.03	0.87
Periaqueductal grey	1.67	0.22		0.39	0.545	0.02	0.898
Piriform cortex	0.29	0.603		1.13	0.309	0.47	0.507
Septum	**7.75**	**0.017**	F>M	0.23	0.644	3.12	0.103
Stria medularis	0.38	0.552		0.01	0.929	0.53	0.482
Stria terminalis	0.71	0.416		0.14	0.712	0.22	0.65
Superior colliculus	0.33	0.575		0.11	0.744	1.02	0.332
Thalamus	0.73	0.409		0.04	0.840	0.65	0.435
Ventricles	11.394	**0.006**	F>M	2.18	0.166	2.19	0.165
Whole brain	1.27	0.282		0.02	0.892	0.57	0.465

Supplementary Table 1. Fractional anisotropy (FA) values for different brain regions after DTI at 3 months post-RT. All significant p values are shown in bold. Sex differences are further denoted by M and F for male and female, respectively.
