# Supplementary material for: Olfactory Memory Impairment Differs by Sex in a Rodent Model of Pediatric Radiotherapy
Source: Front Behav Neurosci. 2018 Aug 2;12:158. doi: 10.3389/fnbeh.2018.00158 (PMC6084003; doi:10.3389/fnbeh.2018.00158)
Supplement: Supplementary file 2 [file Table_2.pdf]

Supplementary Material

Olfactory Memory Impairment Differs by Sex in a Rodent Model of Pediatric Radiotherapy

Emma C. Perez[1,2*], Shaefali P. Rodgers[1], Taeko Inoune[2], Steen E. Pedersen[3,4], J. Leigh Leasure[1,5*], M. Waleed Gaber[2,3*]

*** Correspondence:** Dr. M. Waleed Gaber: gaber@bcm.edu

**Brain region (RD)**	**Sex**			**Radiation**		**Sex*Radiation**	
	**F value**	**p**		**F value**	**p**	**F value**	**p**
Accumbens nucleus	3.24	0.097		2.34	0.152	0.03	0.867
Amygdala	1.39	0.261		3.75	0.077	1.64	0.225
Anterior commissure	0.07	0.789		0.69	0.421	0.34	0.571
Caudate putamen	8.38	**0.013**	M>F	2.14	0.169	0.04	0.840
Cerebellum	0.068	0.798		0.00	1	0.91	0.359
Cingulum	0.62	0.447		0.76	0.402	0.45	0.514
Claustrum	6.66	**0.024**	M>F	2.04	0.179	0.38	0.552
Corpus callosum & external capsule	1.00	0.338		0.38	0.548	0.75	0.405
Dorsal & ventral endopiriform nucleus	1.48	0.247		2.81	0.119	0.13	0.723
Fasiculus retroflexus	4.56	0.054		1.64	0.225	0.09	0.774
Fimbria	0.18	0.675		1.70	0.217	0.00	1
Fornix	7.11	**0.021**	M>F	5.07	**0.044** (RT>)	0.15	0.709
Hippocampus	0.46	0.512		0.58	0.460	0.13	0.727
Hypothalamus	7.10	**0.021**	M>F	2.48	0.142	0.60	0.453
Inferior colliculus	0.31	0.587		0.32	0.580	0.00	1
Internal capsule	4.14	0.065		0.85	0.376	0.48	0.503
Lateral globus pallidus	7.88	**0.016**	M>F	1.48	0.248	0.09	0.776
Mammillothalamic tract	2.57	.135		1.56	0.235	0.17	0.692

Neocortex	0.22	0.651		0.40	0.537	0.03	0.874
Nosebulb	0.66	0.433		0.07	0.796	0.02	0.899
Optic tract	8.58	**0.013**	M>F	0.42	0.528	0.00	1
Periaqueductal grey	2.46	0.143		0.40	0.541	0.54	0.479
Piriform cortex	0.73	0.411		3.59	0.083	1.82	0.202
Septum	2.61	0.132		4.51	0.055	0.12	0.738
Stria medularis	8.22	**0.014**	M>F	0.90	0.362	1.02	0.332
Stria terminalis	10.21	**0.008**	M>F	2.79	0.121	0.47	0.507
Superior colliculus	2.86	0.116		0.32	0.581	0.03	0.868
Thalamus	20.03	**0.001**	M>F	1.95	0.187	0.26	0.622
Ventricles	19.60	**0.001**	M>F	0.76	0.401	2.85	0.117
Whole brain	3.16	0.101		1.80	0.205	0.32	0.580

Supplementary Table 2. Radial diffusivity (RD) values for different brain regions after DTI at 3 months post-RT. All significant p values are shown in bold. Sex differences are further denoted by M and F for male and female, respectively.
